# E2/ER β inhibit ISO-induced cardiac cellular hypertrophy by suppressing Ca^2+^-calcineurin signaling

**DOI:** 10.1371/journal.pone.0184153

**Published:** 2017-09-01

**Authors:** Cheng-Yen Tsai, Wei-Wen Kuo, Marthandam Asokan Shibu, Yueh-Min Lin, Chien-Nam Liu, Yi-Hui Chen, Cecilia-Hsuan Day, Chia-Yao Shen, Vijaya Padma Viswanadha, Chih-Yang Huang

**Affiliations:** 1 Department of Pediatrics, China Medical University Beigang Hospital, Yunlin, Taiwan, ROC; 2 Department of Biological Science and Technology, College of Biopharmaceutical and Food Sciences, China Medical University, Taichung, Taiwan, ROC; 3 Graduate Institute of Basic Medical Science, China Medical University, Taichung, Taiwan; 4 Department of Pathology, Changhua Christian Hospital, Changhua, Taiwan; 5 Department of M-Commerce and Multimedia Applications, Asia University, Taichung, Taiwan; 6 Department of Nursing, Meiho University, Pingtung, Taiwan; 7 Department of Biotechnology, Bharathiar University, Coimbatore, India; 8 Graduate Institute of Chinese Medical Science, China Medical University, Taichung, Taiwan; 9 Department of Biotechnology, Asia University, Taichung, Taiwan; Faculty of Medicine & Health Science, UNITED ARAB EMIRATES

## Abstract

Cardiovascular incidences are markedly higher in men than in pre-menstrual women. However, this advantage in women declines with aging and therefore can be correlated with the sex hormone 17β-Estradiol (E2) which is reported to protect heart cells by acting though estrogen receptors (ERs). In this study we have determined the effect of E2/ERβ against ISO induced cellular hypertrophy in H9c2 cardiomyoblast cells. The results confirm that ISO induced cardiac-hypertrophy by elevating the levels of hypertrophy associated proteins, ANP and BNP and further by upregulating p-CaMKII, calcineurin, p-GATA4 and NFATc3 which was correlated with a significant enlargement of the H9c2 cardiomyoblast. However, overexpression of ERβ and/or administration of E2 inhibited ISO-induced hypertrophy in H9c2 cells. In addition, E2/ERβ also inhibited ISO-induced NFATc3 translocation, and reduced the protein level of downstream marker, BNP. Furthermore, by testing with the calcineurin inhibitor (CsA), it was confirmed that calcineurin acted as a key mediator for the anti-hypertrophic effect of E2/ERβ. In cells treated with calcium blocker (BATPA), the inhibitory effect of E2/ERβ on ISO-induced Ca^2+^ influx and hypertrophic effects were totally blocked suggesting that E2/ERβ inhibited calcineurin activity to activate I-1 protein and suppress PP1, then induce PLB protein phosphorylation and activation, resulting in Ca^2+^ reuptake into sarcoplasmic reticulum through SR Ca^2+^ cycling modification. In conclusion, E2/ERβ suppresses the Ca^2+^ influx and calcineurin activity induced by ISO to enhance the PLB protein activity and SR Ca^2+^ cycling.

## Introduction

According to the World Health Organization statistics, heart disease is the most common cause of disease-related death worldwide. Heart diseases are markedly more common among men than in women. However, incidences of heart disease increases with age, and the increase is sharp among post-menopausal women [1]. Hypertrophy and apoptosis in cardiomyocytes is known to contribute to progression of heart failure, arrhythmias. Angiotensin II (Ang II), β-adrenergic receptor agonist, norepinephrine and other hormones play roles in inducing cardiac hypertrophy [2–4]. In addition, calcium regulates longer-term effects such as hypertrophy or apoptosis by excitation-transcription coupling [5] Under ISO or AngII stimulation, calcium ions activate calcineurin and dephosphorylate p-NFAT3, which leads to the transactivation of ANP and BNP [6,7].

β1-AR is a major subtype of β-adrenergic receptor in cardiomyocytes [8,9]. Activation of β1-AR triggers cAMP/protein kinase A (PKA) signaling activation, inhibiting Ca^2+^ influx to enhance cardiomyocyte contractility-relaxation function. It has been well established that activated PKA phosphorylates phospholamban (PLB) to p-ser^16^-PLB and enables the binding of PLB to SR calcium ATPase (SERCA) on sarcoplasmic reticulum (SR). The binding of PLB with SERCA inhibits SERCA activity and Ca^2+^ uptake into SR storage. It is also known that over-dephosphorylation of PLB by phosphatases such as PP2B and type 1 phosphatase (PP1) dissociates PLB from SERCA resulting in the induction of SERCA activity and Ca^2+^ uptake into SR storage [10,11]. In failing hearts with low contractile function, the levels of phosphorylated PLB are reduced due to down regulation of β-adrenergic receptors and in addition dephopshorylation of PLB is also elevated due to up-regulation in PP1 or PP2B levels [8,12,13]. Both these phenomena contribute to detachment of PLB from SERCA leading to impairment in the performance of Ca^2+^ pump [11,14,15].

Increase in calcium accumulation can increase the activation of Calcineurin which also dephosphorylates NFAT and triggers its translocation from cytosol to nucleus causing hypertrophy. Calcineurin also mediates the dephosphorylation of Bad causing its incorporation on mitochondrial outer membrane thereby triggers apoptosis [16–19].

Previous studies show that the female hormone estrogen modulates calcium-handling proteins, heart function, blood flow; decreases vascular inflammation and arrhythmias and prevents cardiac hypertrophy and myocardial cell apoptosis [20–24]. Three naturally occurring estrogens in women are estrone (E1), 17beta-estradiol (E2) and estriol (E3). When E2 gets translocated into cytosol it interacts with estrogen receptor (ER) which is a member of the nuclear hormone family of intracellular receptors. Its major function is as a transcription factor and regulates various gene expressions. Additionally, two estrogen receptors, the estrogen receptor alpha (ER α) and the estrogen receptor beta (ER β), can interact with 17β-estradiol (E2). Moreover, both ER α and ER β belong to a superfamily of ligand-activated transcription factors that are able to bind to estrogen response elements (ERE) in the regulatory regions of DNA strands [25].

Recently, the estrogen receptors (α&β) were identified to exist in the cardiomyocytes (Kim and Levin, 2006; Deroo and Korach, 2006). It is also known that 17β-Estradiol-estrogen receptor (E2-ER) signaling modulates cAMP-PKA signaling/estrogen receptors (ERs) signaling interaction, to regulate Ca^2+^ influx and thereby protects the heart. 17β-Estradiol (E2) has been reported to prevent development of heart disease via estrogen receptor β (ER β) [26–29]. However, the potential and mechanism of E2/ER β to suppress the effect of cardiac hypertrophy induced by ISO is not fully understood [21,30]. Further, the interaction of E2/ER β with phosphatase in the development of cardiac hypertrophy and apoptosis has to be thoroughly investigated.

Owing to these reasons, we established a Tet-on ER β system in H9c2 myocardial cells and in neonatal rat ventricular myocyte (NRVM) cells, to determine if E2/ER β provide protection against ISO-induced myocardial hypertrophy and apoptosis effects and further investigated the role of phosphatase (PP1 and PP2B) in the effects of E2/ER β.

## Materials and methods

### Cell culture

The rat-derived H9c2 cardiomyoblast cells (CRL-1446) were purchased from American Type Culture Collection (ATCC Cell Biology Collection). Cell cultures were maintained in DMEM supplemented with supplemented with 1% penicillin streptomycin antibiotic-antimycotic mixture, 2 mM glutamine, 1.5 g/L of sodium bicarbonate, 3.5 g/L of Glucose and 10% Cosmic Calf Serum (CCS) at 37°C in a CO_2_ incubator with 5% CO_2_.

### Cardiomyocyte culture

Neonatal cardiomyocytes were isolated and cultured using the commercial Neonatal Cardiomyocyte Isolation System Kit according to manufacturer’s directions (Cellutron Life Technology, Highland Park, NJ). Ventricular cardiomyocytes from one-day-old newborn SD rats were isolated and cultured in DMEM supplemented with 10% fetal bovine serum (FBS), 1% penicillin and streptomycin and 2 mM glutamine. All experimental procedures were performed according to the NIH Guide for the Care and Use of Laboratory Animals. All protocols were approved by the Institutional Animal Care and Use Committee of China Medical University, Taichung, Taiwan.

### Construct Tet-on gene expression system

ERβ cDNA was spliced between the *Bam*HI and *Sal*I restriction site to construct the pTRE-ERβ plasmid. The plasmid was transfected into cells that were pre-transfected by pTet-on plasmid which constitutively expresses rtTA protein that binds to and activates the promoter on pTRE plasmid in the presence of doxycycline or Tet to trigger ERβ expression. Thereby H9c2 cells and neonatal cardiomyocytes were created such that their ERβ expression was control by Dox or Tet.

### Western-blot and immunoprecipitation

The cultured cells were washed with cold PBS and the cells were lysed by lysis buffer (50 mM Tris, pH 7.5, 0.5 M NaCl, 1.0 mM EDTA, pH 7.5, 10% glycerol, 1 mM BME, 1% IGEPAL-630, and proteinase inhibitor cocktail. After 30 min of on ice, the lysate was collected and centrifuged at 12,000g for 15 minutes at 4°C and the proteins in the supernatant was collected and the concentration of the proteins was determined by the Bradford method. An aliquot containing 40 μg of sample was loaded and analyzed by Western blot analysis following previous reports with some changes [31]. Briefly, the proteins were separated by 12% SDS-PAGE and were transferred to PVDF membrane (Millipore, Belford, Massachusetts, USA) and the membranes were blocked with blocking buffer containing 5% non-fat dry milk for an hour. The membranes were then incubated with primary antibodies on a shaker at 4°C overnight. The blots were stained by ECL after the membranes were incubated with horseradish peroxidase-linked anti-rabbit, antimouse, or anti-goat IgG secondary antibodies.

### Nuclear extraction

The nuclear extraction was performed as described previously [32]. The cultured cells were scraped with 3 mL of cold PBS and were centrifuged at 1,000 g for 10 min at 4°C and the pellet was then suspended with 200 μL ice-cold BUFFER-I and followed by adding 20 μL IGEPAL-CA630. The content was vortexed and pelleted by centrifugation at 12,000g for 5 min at 4°C. The pellet was suspended with ice-cold BUFFER-II and mixed by vortexing and left on ice for 30 min and then centrifuged at 15,000 g for 15 minutes at 4°C. The supernatant containing nuclear extract was collected and stored aliquots at -80°C..

### Actin-immunofluorescence (rhodamine–phalloidin)

The cells were stained with (rhodamine–phalloidin) to determine hypertrophic effect and by following the methods described previously [32]. The nucleus was stained blue with 4',6-diamidino-2-phenylindole. The stained cells were visualized and photographed using a Zesis Axioskop and a fluorescence microscope. The cell size was magnified at 200X.

### Immunofluorescence staining

The dishes with H9c2 cells were re-suspended the culture medium and then wash three times with PBS. The fixing, permeabilization and blocking procedures are as previous show in the Cell nuclear & Actin Stain. H9c2 cells were first stained with DAPI (37°C, 30 minutes) and then repeated the washing step. After previous, H9c2 cells were incubated with PBS (2μg/ml 1°Ab, 1% BSA) and prevented from light at 4°C for overnight. Next day, H9c2 cells also were washed with PBS and incubated with PBS (2μg/ml 2°Ab, 1% BSA) and prevented from light at 37°C for 1hour. Finally, the fluorescence images were detected by confocal microscopy.

### Intercellular calcium staining

To seed Tet-on ERβ H9c2 cells on 6 cm^2^ dishes and incubate with DMEM to 60% full. Added Fluo-4 AM into DMEM to final concentration 2μM and incubated for 20 minutes. After incubation, culture medium was removed and cells were washed three times with calcium contained HBSS solution. Replace the culture medium with calcium contained HBSS solution and incubated for 15 minutes. Fluorescence was visualized and photographed with a laser scanning confocal microscope (TCS SP2, Leica)[33].

### Statistical analysis

Averages from at least three independent repeats were obtained for each experimental condition. The Data was statistically evaluated using paired, two-tailed t-test. All averaged data is reported as means (±S.E.).

## Results

### Isoproterenol induces hypertrophy in H9c2 cell via calcineurin related pathway

**Fig 1:**
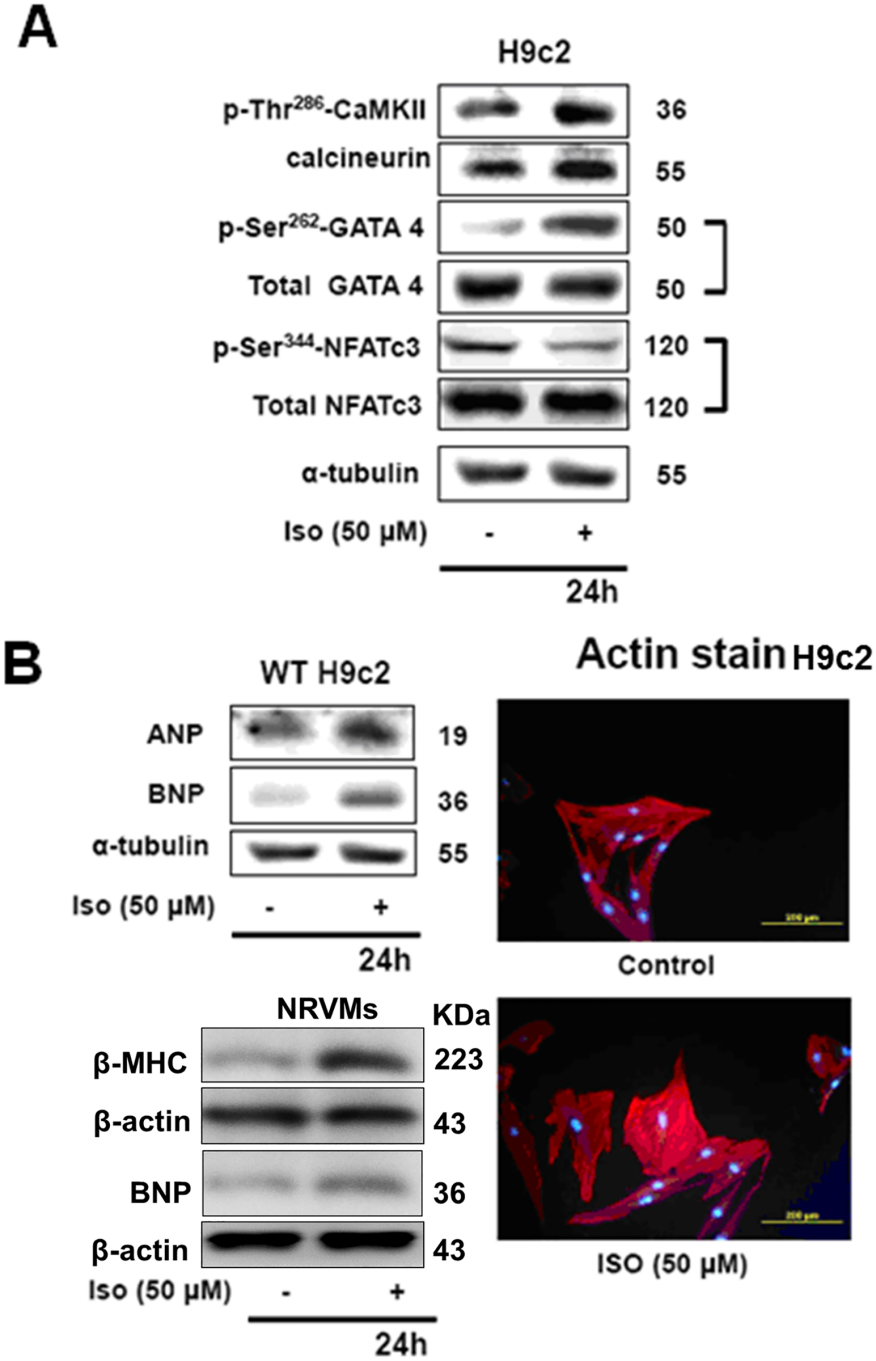
Isoproterenol induces myocardial cell hypertrophy via calcineurin related pathway. H9c2 cells were incubated with ISO (50μM) for 24 hours, and then actin staining and western blotting were performed. A: Representative blots showing levels of p-CaMKII^Ser286^, CaMKII, p-GATA4^Ser262^, GATA4, p-NFATc3^Ser344^, NFATc3, ANP, BNP and α-tubulin detected by Western blotting. B, Western blots showing levels of ANP and BNP proteins in H9c2 cells and β-MHC and BNP levels in NRVMs and fluorescent microscope images show actin stained H9c2 showing hypertrophic effect when treated with ISO.

When H9c2 cells were incubated for 24 h with 50 μM ISO they showed evidences of undergoing hypertrophic effects. Actin staining of the cells show that H9c2 showed increase in the cell size and elongation in the actin filaments indicating the hypertrophic effects. Further confirmation by western blot expression analysis showed the levels of the hypertrophy markers such as brain natriuretic peptide (BNP) and Atrial natriuretic peptide (ANP) were elevated with the 24 h ISO treatment (Fig 1A). The results further showed that levels of calcineurin associated hypertrophic proteins p-CaMKII^Ser286^, p-GATA4^Ser262^ and p-NFATc3^Ser344^ were highly expressed with ISO treatment. NRVM cells also showed an elevation of β-MHC proteins indicating the phenomenon of MHC class switching that is associated with cellular hypertrophic effects caused by β-adrenergic overstimulation. Increase in β-MHC levels in NRVMs was also correlated with increase in the hypertrophic marker BNP. Therefore the results indicate that 50 μM of ISO treatment for 24 h induces calcineurin associated hypertrophy.

### E2/ERβ inhibits ISO-induced cellular hypertrophy in Tet-on ERβ H9c2 myocardial cells

**Fig 2:**
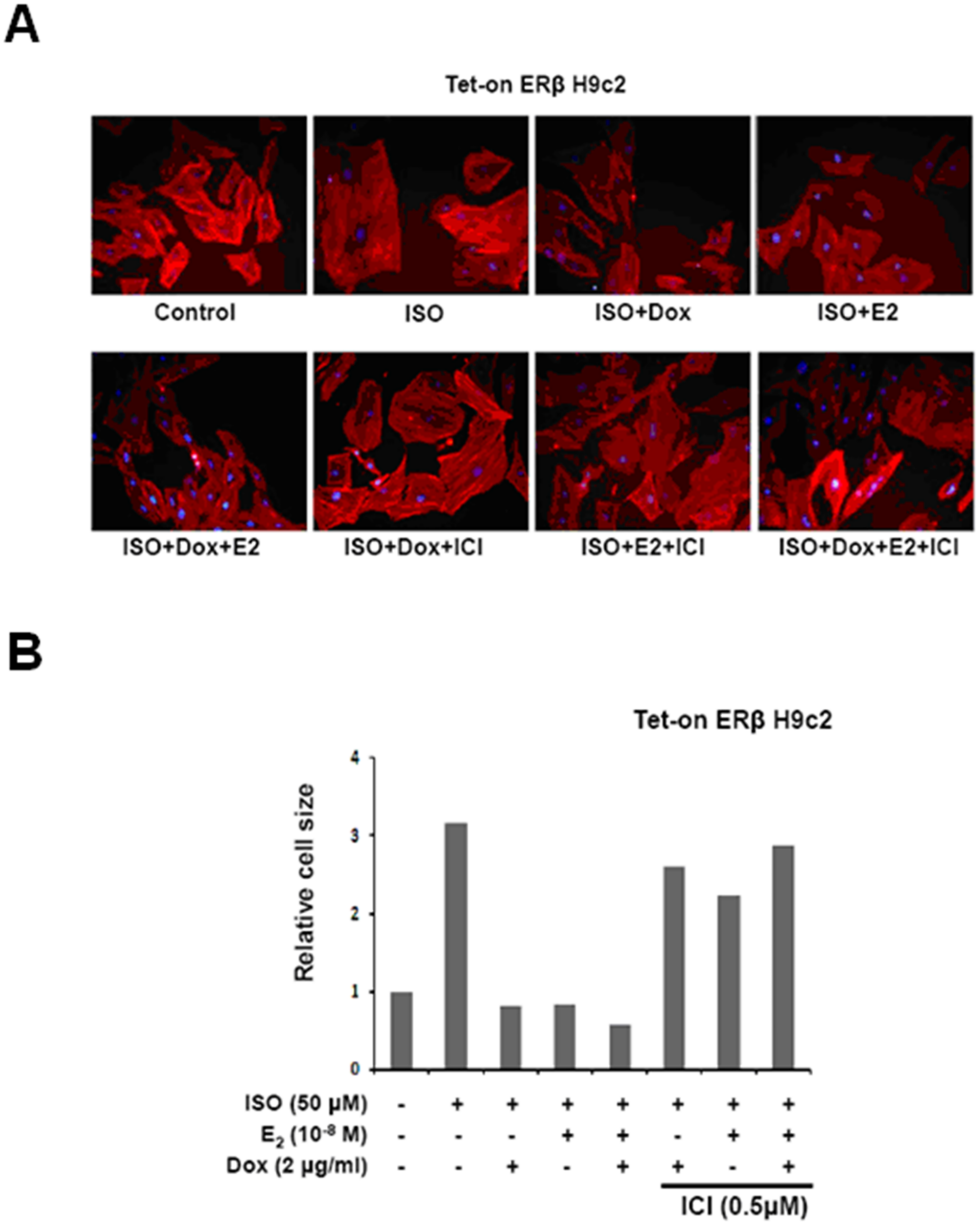
E2/ERβ inhibits ISO-induced cellular hypertrophy in Tet-on ERβ H9c2 myocardial cells. Tet-on/ERα H9c2 cardiacmyobalst cells were incubated with Dox (1μg/ml) and E2 (10^-8^M) in presence or absence of ISO (50μM) and ICI (0.5μM) for 24 hour, then Actin and DAPI double-staining were performed. A: The images were detected by fluorescent microscope. B: The cardiomyoblast cells area were measured from one independent experiment. Mean ± S.D., n = 4.

When H9c2 cells were pretreated with estrogen (E2) or overexpressed with estrogen receptor β (ERβ) prior to 24 h ISO treatment the hypertrophic effects were found to be effectively suppressed as evident from their cell size and actin elongation revealed by actin staining assay. Treatment with E2 or overexpression of E2 retained the cell size to that of normal cells however, the E2 and ERβ effects were inhibited with the pretreatment of (ICI), inhibitor that inhibits estrogen receptor α (ERα) and estrogen receptor β (ERβ). The results (Fig 2A and 2B) therefore point out that E2/ERβ prevents ISO-induced cell hypertrophy in a significant manner.

### E2/ERβ inhibit ISO-induced hypertrophy maker BNP expression in Tet-on ERβ H9c2 myocardial cells

**Fig 3:**
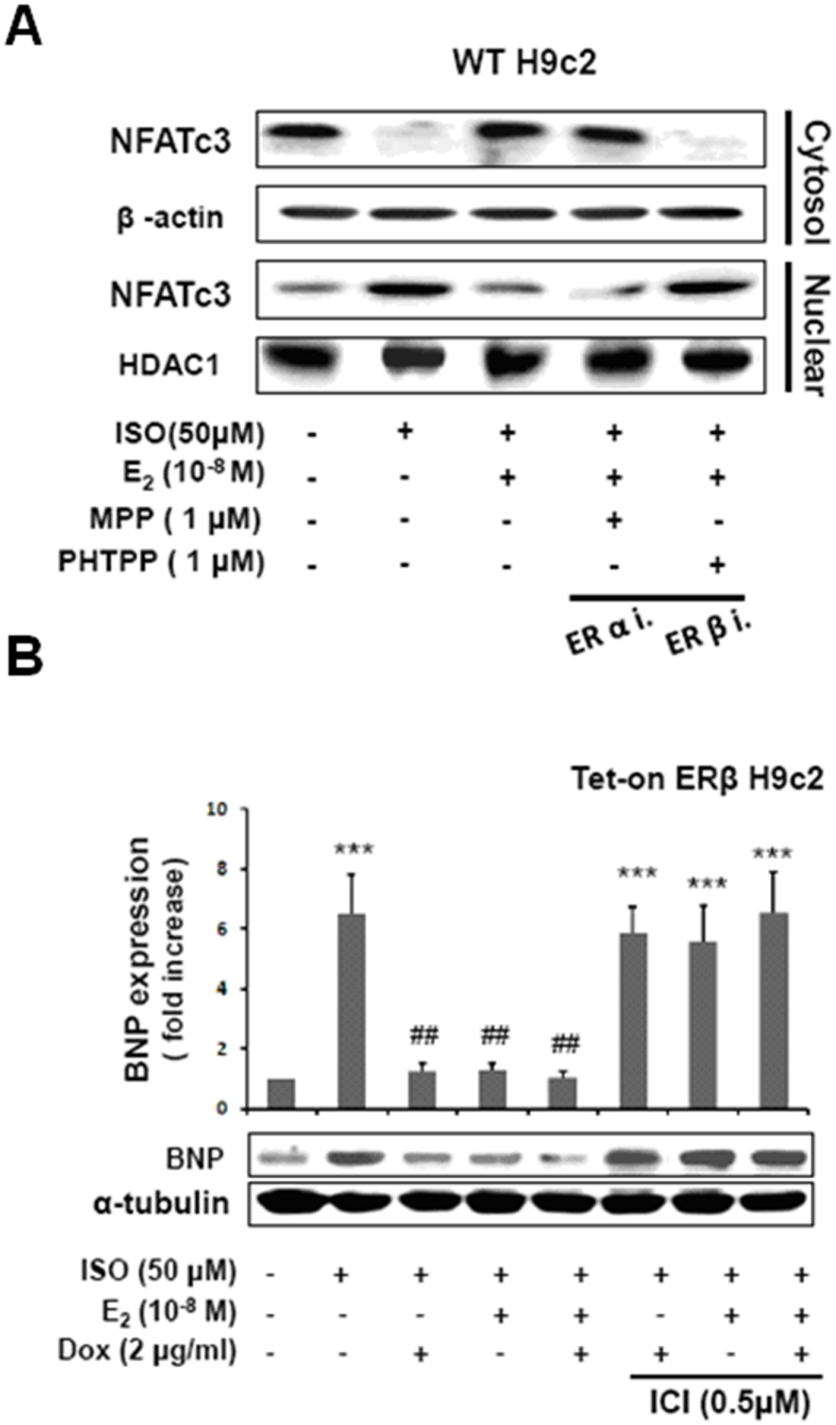
E2/ERβ inhibits ISO-induced NFAT activation in H9c2 myocardial cells. A: Tet-on ERβ H9c2 cells were incubated with E2 (10^-8^M), Dox (2μg/ml), ICI (0.5μM) in the presence of ISO (50μM) for 24 hours, then western blotting was performed, BNP and α-tubulin were detected. B: H9c2 cells were incubated with E2 (10^-8^M), ERα inhibitor MPP (1μM), ERβ inhibitor PHTPP (1μM) in the presence of ISO (50μM) for 24 hours, then cytosol/nuclear isolation assay was performed. To extract cytosolic and nuclear proteins by nuclei extraction assay, then NFATc3, HDAC1 and α-tubulin were detected by Western blot. The statistical results were shown from three independent experiments; mean ± S.D., n = 3. (***p < 0.001 significant differences from ISO group; ##p < 0.01 significant differences from ISO group).

To confirm the previous results that ISO causes hypertrophy in H9c2 cardiomyoblast cells and that estrogen along with ERβ effectively reduces the respective hypertrophy, we analyzed the expression levels of BNP using western blot analysis. Under ISO treatment, the protein expression of BNP in H9c2 cells increased significantly, but was reduced by E2/ERβ administrations. However, the effect of E2/ERβ was abolished by the ER inhibitor (Fig 3A).

### E2/ERβ inhibit ISO-induced NFATc3 nuclear accumulation in H9c2 myocardial cells

Further analysis on the calcineurin mediated hypertrophy associated crucial transcription factor NFATc3 shows that ISO challenge triggers the nuclear localization of NFATc3 however treatment with estrogen (E2) and estrogen receptor β (ERβ) effectively prevented the ISO-induced nuclear accumulation NFATc3 (Fig 3B). Meanwhile in the presence of the ERα inhibitor MPP the effects of E2 pretreatment or ERβ overexpression remained unaltered however when treated with ERβ specific inhibitor PHTPP the inhibition on the NFATc3 nuclear translocation triggered by E2 and ERβ was completely suppressed. Therefore E2 potentially acts through ERβ and not through ERα to suppress NFATc3 activity.

### E2/ERβ inhibits ISO-induced myocardial cell hypertrophy through calcineurin suppression

**Fig 4:**
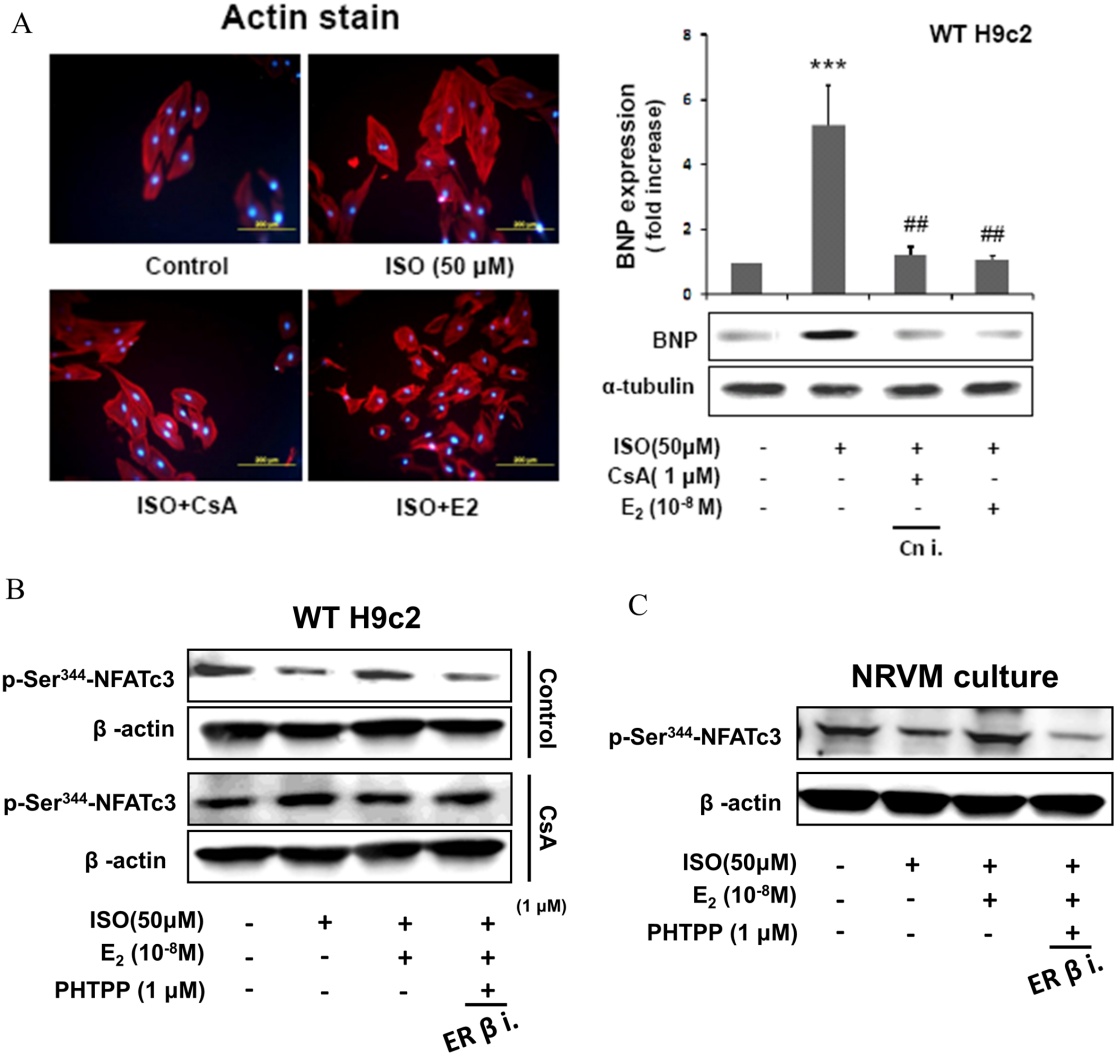
E2/ERβ inhibit ISO-induced myocardial cell hypertrophy through calcineurin suppression. A: H9c2 cells were incubated with E2 (10^-8^M), CsA (1μM) in the presence of ISO (50μM) for 24 hours, then western blotting and actin staining were performed. BNP and α-tubulin were detected by Western blot. The images were detected by fluorescent microscope. The statistical results were shown from three independent experiments; mean ± S.D., n = 3. (***p < 0.001 significant differences from Control group; ##p < 0.01 significant differences from ISO group). B: H9c2 cells were incubated with E2 (10^-8^M), ISO (50μM), PHTPP (1μM) in the presence of CsA (1μM) for 24 hours, then western blotting was performed. p-NFATc3^Ser344^ and β-actin were detected by Western blot. C: NRVM cells were incubated with E2 (10^-8^M), PHTPP (1μM) in the presence of ISO (50μM) for 24 hours. After treatments, then western blotting was performed. p-NFATc3^Ser344^ and β-actin were detected by Western blot.

To identify whether calcineurin participated in isoproterenol-induced hypertrophy signaling pathway, western blotting and actin staining were performed subsequent to administration of calcineurin inhibitor (CsA). The results showed that CsA inhibited ISO induced hypertrophic effects and was similar to the effect of E2 as determined by respective actin staining analysis. The BNP expression was also suppressed in the presence of CsA in ISO treated H9c2 cells. Therefore the results depicts that ISO induced BNP expression and associated cellular hypertrophy in H9c2 cells is mediated through calcineurin (Fig 4A). Further analysis on the involvement of calcineurin on nuclear translocation of active NFATc3 show that the ISO mediated stimulation of pNFATc3 nuclear accumulation and E2/ERβ inhibition were completely neutralized in the presence of CsA. The results observed in the H9c2 cells were also confirmed in NRVM cells (Fig 4B and 4C).

### E2/ERβ inhibits ISO-induced calcineurin activity by modulating SR Ca^2+^ cycling

**Fig 5:**
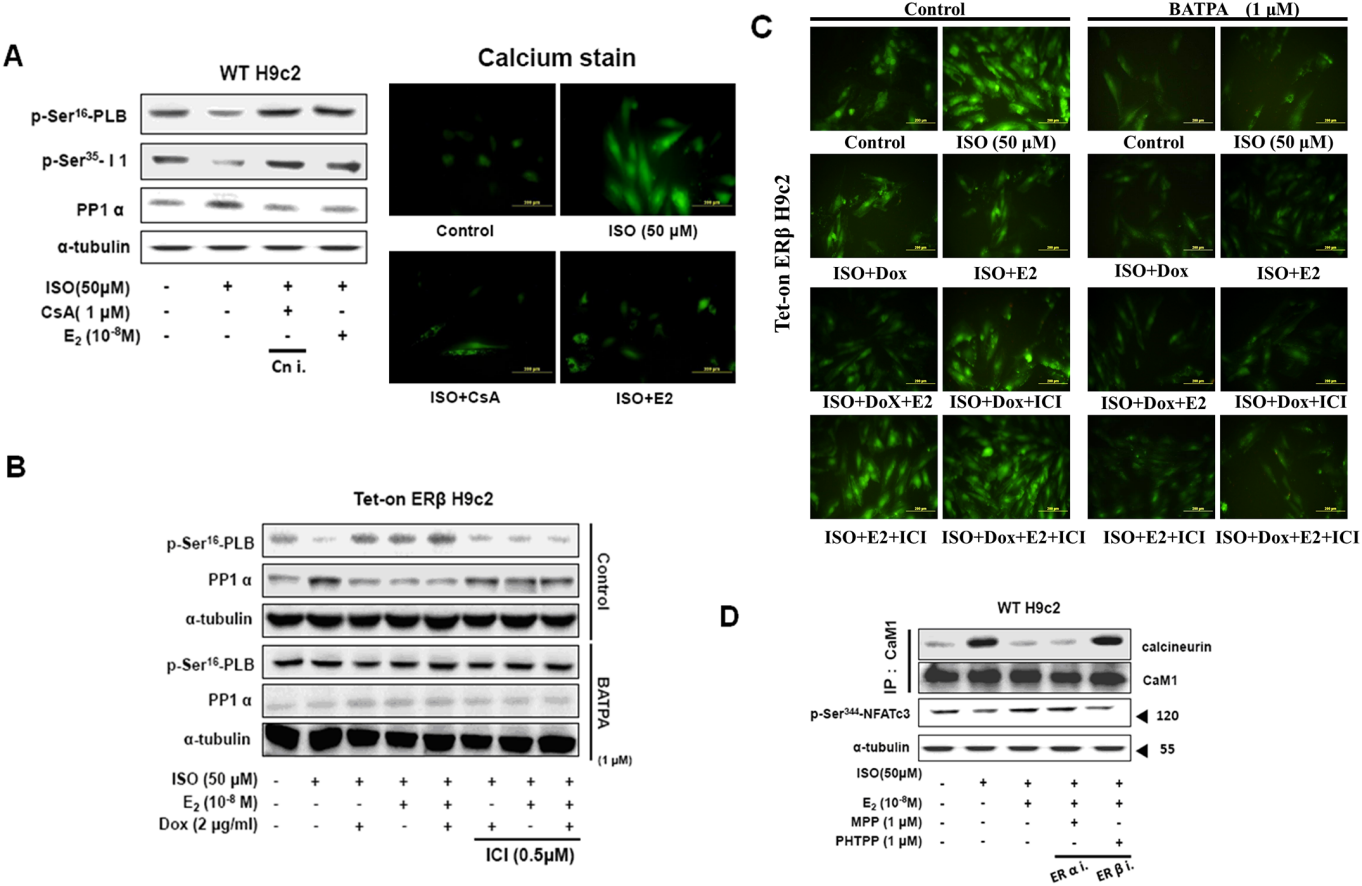
E2/ERβ inhibits ISO-induced calcineurin activity through SR Ca^2+^ cycling modification. A: H9c2 cells were incubated with E2 (10^-8^M), CsA (1μM) in the presence of ISO (50μM) for 24 hours, then western blotting and calcium staining were performed. PP1α, p-PLB^Ser16^, p-I1^Ser35^ and α-tubulin were detected by Western blot. The images were detected by fluorescent microscope. B, C: Tet-on ERβ H9c2 cells were incubated with E2 (10^-8^M), Dox (2μg/ml), ICI (0.5μM), ISO (50μM) in the presence of BATPA (1μM) for 24 hours, then western blotting and fluo-4AM calcium staining were performed. PP1α, p-PLB^Ser16^ and α-tubulin were detected by Western blot. The images were detected by fluorescent microscope. D: H9c2 cells were incubated with E2 (10^-8^M), MPP (1μM), PHTPP (1μM) in the presence of ISO (50μM) for 24 hours, then immunoprecipitation assay was performed. CaM, calcineurin, p-NFATc3^Ser344^ and α-tubulin were detected by Western blot.

Further analysis on the effect of ISO on the PLB activity associated with SR Ca^2+^ cycling showed that ISO triggered PLB activity was correlated with PP1α activation and elevated dephosphorylated I-1^Thr34^ levels (Fig 5A). The ISO induced changes in pPLB, pI-1 and PP1α were suppressed by CsA treatment and the modulation were accompanied with the corresponding changes in the calcium levels as determined from the calcium staining of the H9c2 cells. ISO induced Ca^2+^ influx to inhibit I-1 protein and activate PP1 and further induced PLB protein dephosphorylation however, E2/ERβ inhibited the ISO-induced effects on Ca^2+^ influx to activate I-1 protein, suppress PP1 and induce PLB protein phosphorylation and activation. However, the effects of E2/ERβ were totally blocked by ER inhibitor, ICI. Further, in the presence of BAPTA, a Ca^2+^ chelator, the effect of ISO and the influence of E2/ER were silenced (Fig 5B and 5C). Therefore the results show that E2/ERβ inhibits ISO-induced Ca^2+^ influx to stabilize PLB activity via blocking SR Ca^2+^ cycling. Moreover, co-IP assay with CaM1 and calcineurin show that calcineurin mediated hypertrophy induced by ISO involves CaM-calcineurin binding to activate calcineurin, but E2/ERβ inhibits the ISO-induced CaM-calcineurin binding to suppress calcineurin (Fig 5D). Administration of ERα specific inhibitor MPP and ERβ specific inhibitor PHTPP showed that ERβ and not ERα is involved in the antihypertrophic effects against ISO induced cardiac hypertrophy.

## Discussion

The sympathetic nervous system (SNS) plays an integral role in regulating cardiac function. Evidence suggests that enhanced SNS activation can have harmful effects on the heart, resulting in heart failure [34,35]. Hence, SNS inhibition in cardiomyopathy provides a therapeutic direction for the administration of β-adrenergic receptor (β-AR) blockers in patients with heart failure. In clinical observations, therapeutic interventions using β-AR blockers efficiently improve the cardiac contractility and greatly improve the heart failure prognosis [36,37]. The incidence and mortality from cardiovascular disease (CVD) have been found to be low in premenopausal women but significantly increased in menopausal women, suggesting that estrogen acts as a protector for the cardiovascular system [38].

Previous studies demonstrated that there are many important molecules that participate in the cardiac remodeling mechanism to help the heart increase its’ out-put. However, over abundant expression of some molecules may induce the heart into irreversible pathological hypertrophy as published in clinical reports such as: PP1, PP2A, and calcineurin [39–41]. This study found that isoproterenol (50 μM) induces cardiac hypertrophy through calcineurin, as observed by western blotting, actin staining and NFAT translocation. However, E2/ERβ can reduce the isoproterenol induced myocardial cell hypertrophy effect. As we know, calcium is the upstream signal for cardiac hypertrophy. It has also been reported that over-dephosphorylation of PLB by type 1 phosphatase (PP1) leads to SERCA inhibition caused heart failure in human patients. PLB over-dephosphorylation will increase the Ca^2+^ level in cytosol and induce CaM-calcineurin/NFAT signaling activation [42]. This study found that E2/ERβ can reduce isoproterenol-induced calcineurin activity through SR Ca^2+^ cycling modification, as observed by western blotting, calcium staining and co-IP assay. E2/ERβ inhibits isoproterenol induced myocardial cell hypertrophy.

**Fig 6:**
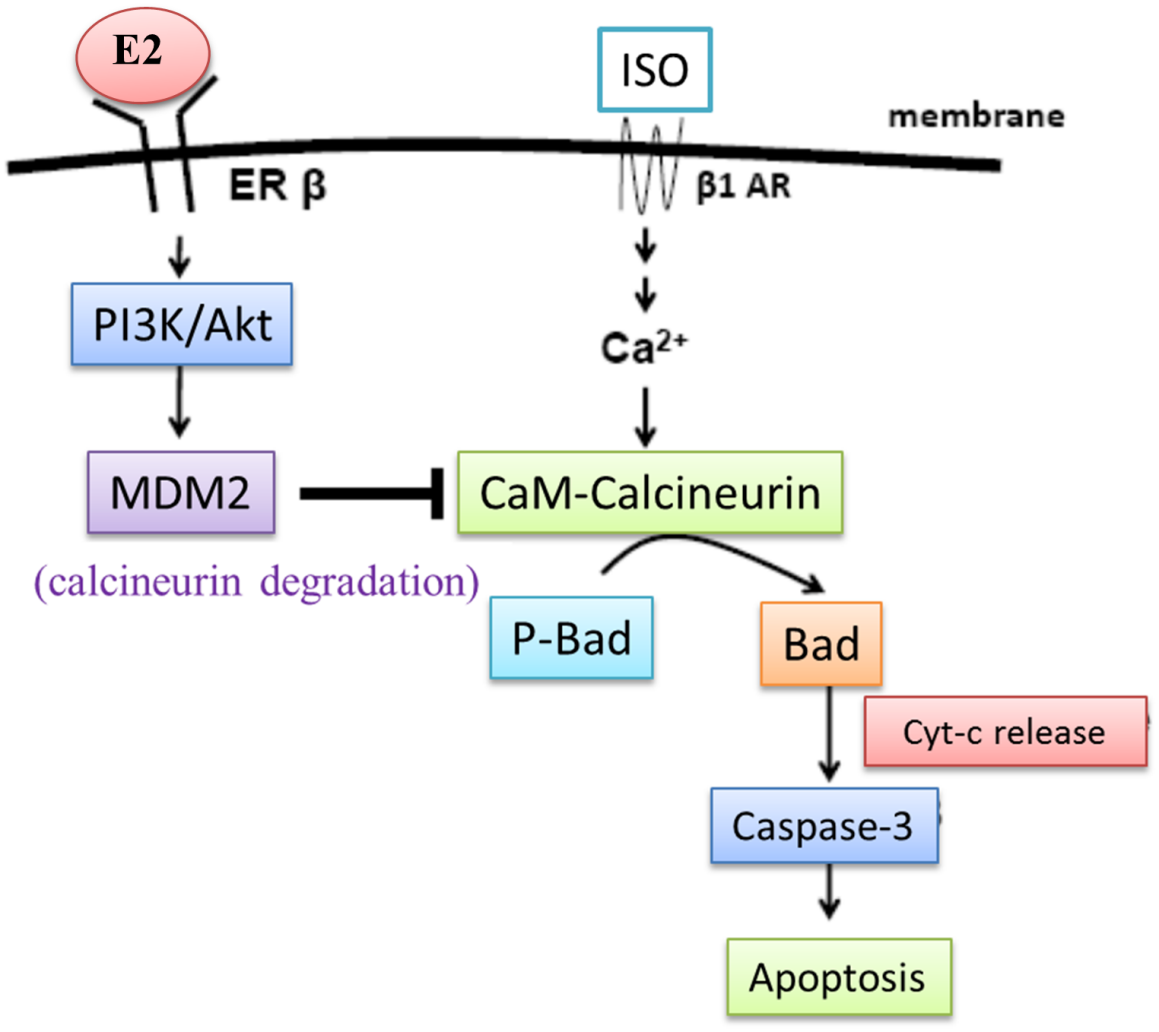
E2/ER β inhibit ISO-induced myocardial cell hypertrophy through Ca^2+^-calcineurin signaling suppression.

The occurrence and susceptibility to cardiovascular disease (CVD) in premenopausal women are generally very low but are significantly higher among menopausal women. Therefore estrogen potentially acts as a protector of cardiovascular system against various stresses. In this study we investigated the cardio-protective effects and mechanisms provided by E2 and ERβ in myocardiac cells exposed to the β-adrenergic receptor agonist, Isoproterenol. Calcineurin inhibitor (CsA) was applied in H9c2 and neonatal rat ventricular myocyte (NRVM), and found that calcineurin was the key mediator for E2/ERβ function. In cells treated with calcium blocker (BATPA), the E2/ERβ inhibited ISO-induced Ca^2+^ influx and hypertrophic effects were totally blocked, suggesting that E2/ERβ inhibited calcineurin activity to activate I-1 protein and suppress PP1, then inducing PLB protein phosphorylation and activation, resulting in Ca^2+^ reuptake into the sarcoplasmic reticulum through SR Ca^2+^ cycling modification. This study clearly shows that the cardio-protective effects and mechanisms of E2 and ERβ are involved in mediating myocardial cell calcineurin activity (Fig 6). In conclusion, we found that E2/ERβ inhibits ISO-induced myocardial cell hypertrophy through Ca^2+^-calcineurin signaling suppression. E2 effectively prevented ISO-induced cell hypertrophy via calcineurin activity dependent ERβ.
